# C-Terminal Lysine Residue of Pneumococcal Triosephosphate Isomerase Contributes to Its Binding to Host Plasminogen

**DOI:** 10.3390/microorganisms11051198

**Published:** 2023-05-04

**Authors:** Satoru Hirayama, Takumi Hiyoshi, Yoshihito Yasui, Hisanori Domon, Yutaka Terao

**Affiliations:** 1 Division of Microbiology and Infectious Diseases, Niigata University Graduate School of Medical and Dental Sciences, Niigata 951-8514, Japan; 2 Division of Periodontology, Niigata University Graduate School of Medical and Dental Sciences, Niigata 951-8514, Japan; 3 Center for Advanced Oral Science, Niigata University Graduate School of Medical and Dental Sciences, Niigata 951-8514, Japan

**Keywords:** lysine residue, plasmin, plasminogen, *Streptococcus pneumoniae*, triosephosphate isomerase

## Abstract

The main causative agent of pneumonia, *Streptococcus pneumoniae*, is also responsible for invasive diseases. *S. pneumoniae* recruits human plasminogen for the invasion and colonization of host tissues. We previously discovered that *S. pneumoniae* triosephosphate isomerase (TpiA), an enzyme involved in intracellular metabolism that is essential for survival, is released extracellularly to bind human plasminogen and facilitate its activation. Epsilon-aminocaproic acid, a lysine analogue, inhibits this binding, suggesting that the lysine residues in TpiA are involved in plasminogen binding. In this study, we generated site-directed mutant recombinants in which the lysine residue in TpiA was replaced with alanine and analyzed their binding activities to human plasminogen. Results from blot analysis, enzyme-linked immunosorbent assay, and surface plasmon resonance assay revealed that the lysine residue at the C-terminus of TpiA is primarily involved in binding to human plasminogen. Furthermore, we found that TpiA binding to plasminogen through its C-terminal lysine residue was required for the promotion of plasmin activation by activating factors.

## 1. Introduction

Pneumococcal infections continue to be a global epidemic, posing a significant threat to children and the elderly with high incidence and mortality rates [1,2,3]. *Streptococcus pneumoniae*, the primary causative agent of pneumonia, is a gram-positive diplococcus that inhabits the human nasopharynx and commonly causes community-acquired pneumonia, otitis media, and sinusitis. In addition to these noninvasive diseases, it is also known to cause invasive diseases such as bacteremia, sepsis, and meningitis [4,5].

To achieve invasiveness in the host, *S. pneumoniae* utilizes human plasminogen by recruiting it to the bacterial surface and aiding in the breakdown of physiological barriers, such as the extracellular matrix (ECM) of host cells. Plasminogen bound to the bacterial cell surface is converted by human tissue-type plasminogen activator (tPA) or urokinase-type plasminogen activator (uPA) into plasmin, a serine protease with protein-degrading activity [6,7]. Several plasminogen-binding proteins have been identified in *S. pneumoniae*.

We recently identified triosephosphate isomerase (TpiA) as a novel plasminogen-binding protein in *S. pneumoniae* [8]. TpiA is a protein with a molecular weight of approximately 27,000 Da that exists as an enzymatic dimer [9]. It is present in all organisms and is one of the glycolytic enzymes that catalyzes the reversible isomerization of glyceraldehyde-3-phosphate and dihydroxyacetone phosphate [9,10,11]. Pneumococcal TpiA binds to human plasminogen and promotes activator-mediated plasminogen activation [8]. The binding of TpiA to plasminogen is suggested to involve lysine residues in TpiA, as it was dose-dependently inhibited by the addition of epsilon-aminocaproic acid (EACA), a lysine analogue [8].

In the present study, we generated site-specific mutants of pneumococcal TpiA by substituting each of the 18 lysine residues with alanine. Additionally, we prepared a recombinant mutant in which all three lysine residues located in a concentrated region were substituted with alanine. We analyzed the binding characteristics of the 19 site-specific mutant recombinants to plasminogen. Among them, the recombinant TpiA K252A mutant exhibited a significant decrease in binding with plasminogen, suggesting that the C-terminal lysine residue is involved in this binding characteristic.

## 2. Materials and Methods

### 2.1. Bacterial Strain and Growth Media

We expressed and produced His-tagged rTpiA proteins with site-specific amino acid substitutions using *Brevibacillus choshinensis* strain HPD31-SP3. Transformants of this strain were cultured on MTNm plates (10 g/L glucose, 10 g/L phytone peptone, 2 g/L yeast extract, 5.75 g/L Erlich bonito extract [35%], 10 mg/L MnSO_4_·4H_2_O, 10 mg/L FeSO_4_·7H_2_O, and 1 mg/L ZnSO_4_·7H_2_O, with the pH adjusted to 7.0 and supplemented with 15 g/L agar, 20 mM MgCl_2_, and 50 µg/mL neomycin) or in TMNm (MTNm without agar and MgCl_2_) or 2SYNm broths (20 g/L glucose, 40 g/L Bacto soytone, 5 g/L Bacto yeast extract, and 0.15 g/L CaCl_2_·2H_2_O, with the pH adjusted to pH 7.2, and supplemented with 50 µg/mL neomycin).

### 2.2. Construction of the Brevibacillus Strains Producing rTpiA

The off-the-shelf *Brevibacillus* cloning system (Takara Bio, Kusatsu, Shiga, Japan) was used to construct *S. pneumoniae* D39 rTpiA proteins with site-specific amino acid substitutions, as described in our previous study [8]. To introduce site-specific mutations into the *tpiA* gene, an inverse polymerase chain reaction (PCR) was performed using the primers shown in Table 1 with plasmid pBIC2-tpiA [8], where the open reading frame of the *tpiA* gene, except for the start codon, was inserted as the template. PCR was performed using KOD One PCR Master Mix (Toyobo, Osaka, Japan), according to the manufacturer’s instructions. To self-ligate the PCR fragments, aliquots of diluted PCR-amplified products were treated with T4 polynucleotide kinase (Takara Bio) and Ligation-High version 2 (Toyobo) and incubated at 16 °C for at least 2 h. Thereafter, *B. choshinensis* HPD31-SP3 was transformed with the self-ligating plasmid, and transformants were selected on MTNm plates. Colonies of the resultant transformants were inoculated into 2SYNm broth and cultured overnight at 37 °C with shaking at 120 rpm. Bacterial cells were collected, and plasmids were extracted using a QIAprep Spin Miniprep Kit (QIAGEN, Hilden, Germany). The DNA inserted into the plasmid was sequenced by Eurofins Genomics (Tokyo, Japan) using the primers for sequencing (Table 1).

### 2.3. Purification of rTpiA

Each rTpiA protein with a His-tag from the *Brevibacillus* culture supernatant was purified as described in our previous study [8]. *B. choshinensis* HPD31-SP3 expressing His-tagged rTpiA was inoculated and cultured in TMNm broth at 32 °C for 64 h with shaking at 120 rpm. During the stationary phase, the culture supernatant was harvested by centrifugation (12,000× *g* for 20 min) at 4 °C. Thereafter, the supernatant was filtered through a 0.45-µm polyvinylidene fluoride (PVDF) filter (Merck Millipore, Darmstadt, Germany). As pretreatment for the purification of His-tagged rTpiA, Ni Sepharose 6 Fast Flow beads (Cytiva, Marlborough, MA, USA) were washed and resuspended in binding buffer (20 mM NaHPO_4_, pH 7.4, 0.5 M NaCl, and 40 mM imidazole) to produce a 50% slurry. The prepared slurry (1 mL) was added to a dedicated chromatography column (Bio-Rad, Hercules, CA, USA), and the flow-through was discarded. The supernatant was supplemented with NaCl and imidazole until final concentrations of 0.5 M and 40 mM, respectively, were achieved. Next, <10 mL of the supernatant was added to the column using a stopper. The supernatant was incubated with Ni Sepharose 6 Fast Flow beads in a column for 1 h at 25 °C with rotation. After discarding the flow-through, the column was gently washed four times with binding buffer. The targeted proteins that were captured in the column via the interaction between the His-tag and Ni were extracted four times using 500 μL of elution buffer (20 mM NaHPO_4_, pH 7.4, 0.5 M NaCl, and 500 mM imidazole). If necessary, the purified rTpiA solution was desalted, and the solvent was replaced with phosphate-buffered saline (PBS) using PD-10 columns (GE Healthcare, Chicago, IL, USA).

### 2.4. SDS-PAGE and Far-Western Blotting

SDS-PAGE and far-Western blot analyses were conducted according to a previously described formula [8,12,13], with some modifications. Each site-specific substituted rTpiA (400 ng) prepared in PBS was subjected to SDS-PAGE on a 12.5% polyacrylamide gel (ATTO, Tokyo, Japan), and the separated proteins were stained with Coomassie Brilliant Blue (CBB). To investigate the binding activity of each substituted rTpiA to human plasma plasminogen (Sigma-Aldrich, Darmstadt, Germany), far-Western blotting was performed as previously described [8,14], with some modifications. rTpiA (200 ng) and bovine serum albumin (BSA, Sigma-Aldrich) were separated by SDS-PAGE, and the resultant electrophoresis patterns were transferred onto PVDF membranes (Merck Millipore). The transferred membranes were blocked by incubating them with 5% BSA in Tris-buffered saline containing 0.05% Tween 20 (TBST). After washing with TBST, the blocked membranes were incubated for 90 min with 30 µg/mL plasminogen in a blocking reagent at 25 °C. The plasminogen, which binds with proteins on membranes, was probed with rabbit polyclonal antibody against plasminogen (GeneTex, Irvine, CA, USA) in a blocking reagent at a dilution ratio of 1:5000. A horseradish peroxidase (HRP)-conjugated secondary antibody (goat anti-rabbit IgG; Cell Signaling Technology, Beverly, MA, USA) was used at a dilution ratio of 1:3000. Following the addition of the substrate against HRP (ECL Select Western Blotting Detection Reagent; Cytiva), chemiluminescence was visualized and analyzed using an ImageQuant LAS-4000 mini (Fujifilm, Tokyo, Japan).

### 2.5. Enzyme-Linked Immunosorbent Assay (ELISA)

To measure the binding activity of each site-specific substituted rTpiA to human plasminogen, we performed an ELISA based on standard methods [8,13], with some modifications. Each substituted rTpiA was diluted in an ELISA-coating buffer (Na_2_CO_3_ 1.59 g/L, NaHCO_3_ 2.93 g/L, and NaN_3_ 0.2 g/L in water, at pH 9.6) and dispensed into 96-well Half Area Clear Flat Bottom Polystyrene High Bind Microplates (Corning, Corning, NY, USA) at 1 µg/well. Thereafter, the assay plates were incubated overnight at 4 °C. The reacted liquid in the wells was discarded, and 1% skim milk in PBS supplemented with 0.05% Tween 20 (PBST) was added to the wells at 37 °C for 2 h for blocking. The wells were washed with PBST, 1 µg/well of human plasminogen was added, and the plate was incubated at 37 °C for 1 h. Secondary rabbit polyclonal antibodies against plasminogen (GeneTex, Irvine, CA, USA), which were diluted 1:1000 in 0.5% skim milk in PBST, were added after washing with PBST. Following incubation at 37 °C for 1 h, the washed plate was reacted with an alkaline phosphatase (AP)-linked anti-rabbit IgG (H + L) (Bethyl Laboratories, Montgomery, TX, USA), which was diluted 1:5000 in 0.5% skim milk in PBST, and then the assay plate was incubated at 37 °C for 1 h. After washing with PBST, 3 g/L of disodium *p*-nitrophenylphosphate hexahydrate dissolved in diethanolamine buffer (diethanolamine 9.7 mL/L, NaN_3_ 0.2 g/L, and MgCl_2_·6H_2_O 0.1 g/L in water, at pH 9.6) was added into each well. The wells were chemically reacted at 37 °C, and the absorbance at 405 nm (A_405_) was measured using a microplate spectrophotometer (Multiskan FC; Thermo Fisher Scientific, Waltham, MA, USA).

### 2.6. Evaluation of Protein Binding Activity by Surface Plasmon Resonance

Real-time analysis of biomolecular interactions between rTpiA proteins and human plasminogen was performed using surface plasmon resonance (SPR)-based techniques, as previously described [8,15,16,17], with some modifications. SPR measurements were performed using the Biacore X100 system (GE Healthcare). Wild-type rTpiA and site-specific amino acid-substituted rTpiA K252A were diluted in 10 mM sodium acetate buffer (pH 4; Cytiva) to a concentration of 50 μg/mL and thereafter immobilized on a Series S Sensor Chip CM5 (Cytiva) using an amine coupling kit (Cytiva) according to the manufacturer’s instructions. Human plasminogen was diluted from 6.25 nM to 100 nM in running buffer (100 mM HEPES, pH 7.4, containing 150 mM NaCl, 3 mM EDTA, and 0.005% surfactant P20; Cytiva) and injected into the flow cell with a contact time of 120 s. The flow rate was maintained at 10 μL/min and 20 μL/min for immobilization and analysis, respectively. The surface of the sensor chip was regenerated by washing it with 50 mM HCl. The data were analyzed using the Biacore X100 evaluation software (GE Healthcare).

### 2.7. Plasminogen Activation

Plasminogen was activated to form plasmin using tPA according to a previously reported method [8,17,18], with some modifications, to investigate the effect of rTpiA proteins on plasminogen activation. All reagents and proteins were prepared in PBS (pH 7.4). Human plasminogen (2 μg in 80 µL PBS) and 5–40 pmol of rTpiA proteins or BSA (in 10 µL PBS) were mixed and incubated at 37 °C for 30 min in a 96-well microtiter plate. Thereafter, 10 µL of recombinant human tPA (NKMAX, Sungnam, Republic of Korea) at a concentration of 10 µg/mL and 100 µL of 0.45 mM S-2251 chromogenic substrate (DiaPharma Group, West Chester, OH, USA) were added to the reacted wells and incubated at 37 °C. The release of chromogenic *p*-nitroaniline upon the degradation of S-2251 by the generated plasmin was monitored by measuring A_405_ sequentially using a microplate spectrophotometer (Multiskan FC).

### 2.8. Statistical Analysis

All statistical analyses were conducted using Prism 8 software version 8.4.3 (GraphPad Software, La Jolla, CA, USA). Statistical significance was defined as *p* < 0.05.

## 3. Results

### 3.1. rTpiA Proteins with Site-Specific Substitution of Lysine Residues with Alanine Residues

**Figure 1:**
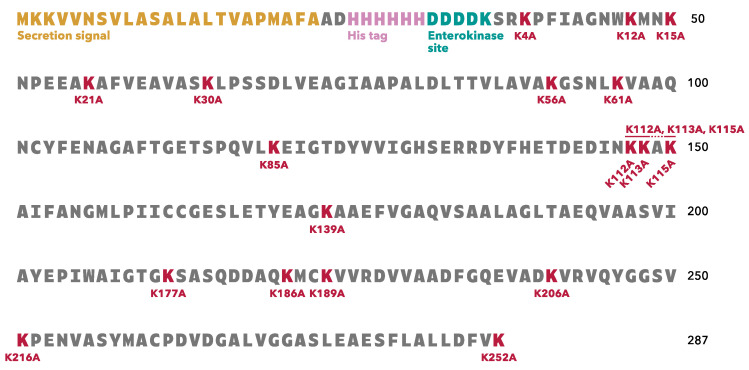
Amino acid sequence of rTpiA expressed by *B. choshinensis* in this study. The rTpiA contains a secretion signal, a His tag, and an enterokinase site.

To clarify which lysine residue in *S. pneumoniae* TpiA is responsible for binding to human plasminogen, we prepared 19 different rTpiA proteins with site-specific amino acid substitutions, in which lysine residues were replaced with alanine residues (Figure 1). The amino acid sequence of *S. pneumoniae* TpiA contains 18 lysine residues, three of which are located close to each other. We prepared site-specific substituted rTpiA proteins in which each lysine residue was replaced with an alanine, as well as a substituted rTpiA in which all three closely located lysines were replaced with alanines.

### 3.2. Binding of rTpiA Proteins with Site-Specific Substitutions to Human Plasminogen

**Figure 2:**
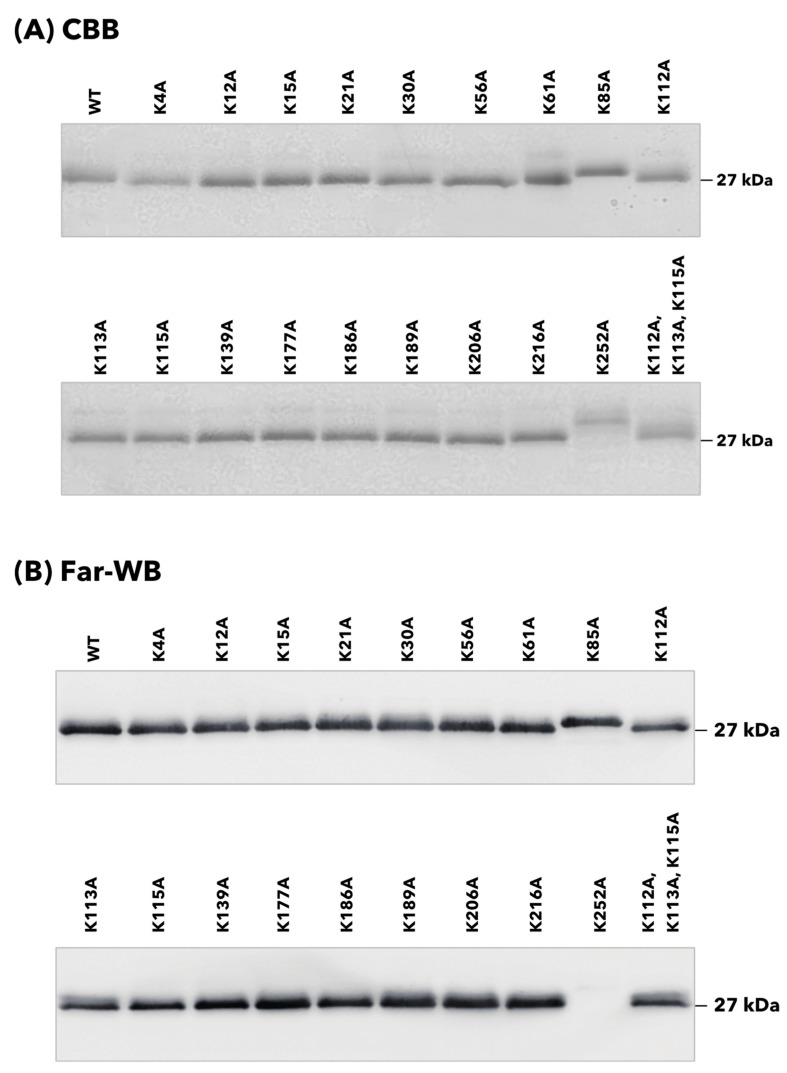
Binding analysis of site-specific amino acid-substituted rTpiA proteins to plasminogen by blot analysis. (**A**) Detection of substituted rTpiA proteins by SDS-PAGE and CBB staining. Each protein (400 ng) was subjected to SDS-PAGE and stained with CBB. The original, uncropped image is shown in Supplementary Figure S1. (**B**) Binding analysis of substituted rTpiA proteins to plasminogen by far-Western blotting. The detection of chemiluminescence from the enzymatic activity of HRP was performed with exposure times of 10 min.

The binding properties of 20 rTpiA proteins, including the wild-type rTpiA, to human plasminogen were qualitatively analyzed. Each rTpiA was subjected to SDS-PAGE analysis and stained with CBB to confirm that each band was approximately 27 kDa (Figure 2A). In the K85A and K252A mutants of rTpiA, the bands were slightly shifted upward. The cause of this is not clear, but several instances have been reported in which a single amino acid substitution can result in fluctuations in the swim position in SDS-PAGE [19,20,21]. At least we have confirmed that the DNA sequence is correct in all rTpiA. Plasminogen was applied to each rTpiA protein, and the bound plasminogen was detected by far-Western blotting; no clear band was observed in the substituted rTpiA K252A (Figure 2B).

**Figure 3:**
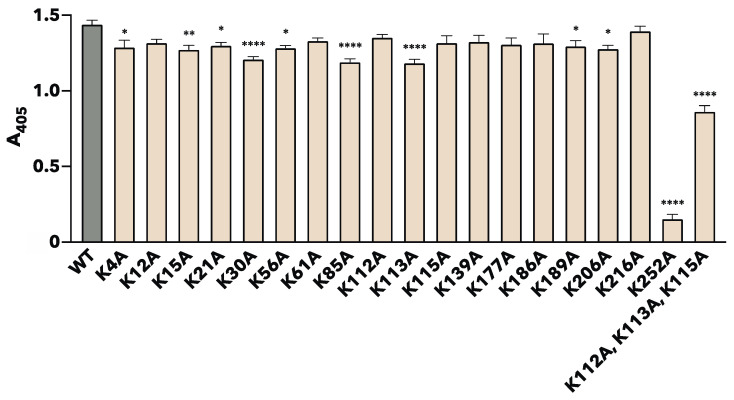
Binding analysis of site-specific amino acid-substituted rTpiA proteins to plasminogen by ELISA. After the addition of plasminogen to the wells of each rTpiA protein-coated microtiter plate, the bound plasminogen was detected using an anti-plasminogen antibody and an AP-conjugated secondary antibody. This result was measured 60 min after substrate addition. Error bars indicate the standard error (*n* = 9). Asterisks indicate significant differences from the WT group (* *p* < 0.05, ** *p* < 0.01, **** *p* < 0.0001, Dunnett’s multiple comparisons test).

The binding properties of each site-specific substituted rTpiA to plasminogen were quantitatively measured in two ways. Plasminogen was added to microtiter plates coated with each rTpiA protein, and the amount of bound plasminogen was indirectly quantified using ELISA (Figure 3). Most of the substituted rTpiA showed significantly reduced binding to plasminogen compared to that of the wild-type rTpiA. Among them, the substituted rTpiA K252A showed significantly lower binding to plasminogen than the other substituted rTpiA proteins (*p* < 0.0001).

**Figure 4:**
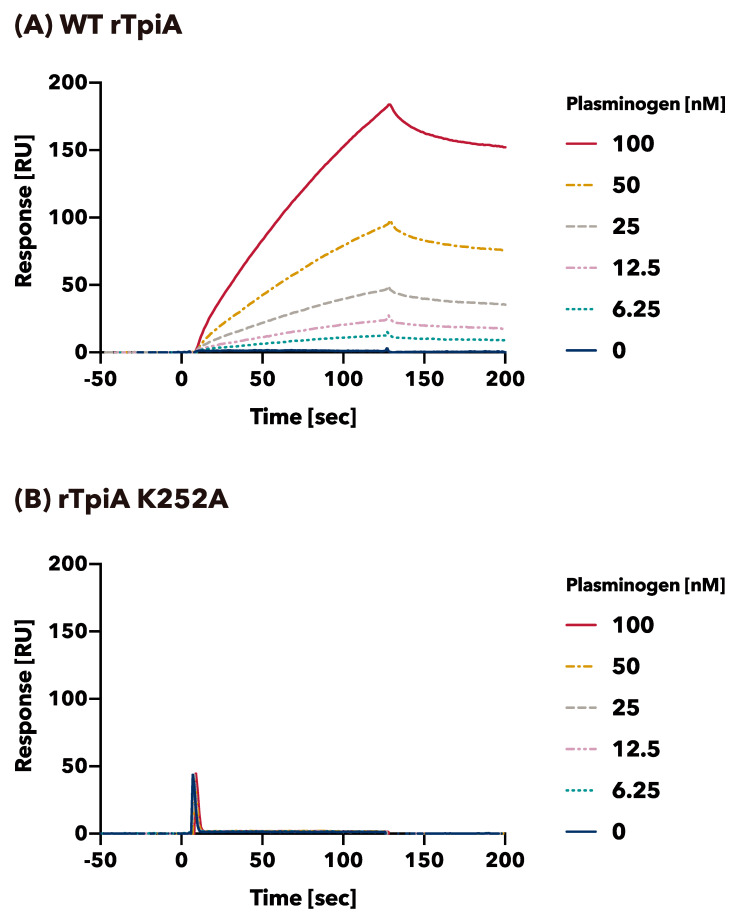
Binding activity of wild-type rTpiA and the site-specific amino acid substituted rTpiA K252A to plasminogen as measured by SPR. The binding of plasminogen to wild-type rTpiA (**A**) or substituted rTpiA K252A (**B**) was analyzed. The experiments were conducted at least three times, with results showing a similar trend. The results of the other two experiments performed using the identical chip are shown in Supplementary Figure S2.

The binding profiles of plasminogen to wild-type rTpiA and substituted rTpiA K252A were quantitatively analyzed by SPR (Figure 4). Human plasminogen is bound to wild-type rTpiA mounted on the biosensor in a dose-dependent manner (Figure 4A). The apparent binding rate (*k_a_*), dissociation rate (*k_d_*), and dissociation constant (K_D_) of plasminogen and wild-type rTpiA were *k_a_* = 4.34 × 10^3^ 1/Ms, *k_d_* = 3.75 × 10^−3^ 1/s, and K_D_ = 8.65 × 10^−7^ M, respectively. In contrast, plasminogen showed little binding to the substituted rTpiA K252A at the same dose (Figure 4B), and therefore, *k_a_*, *k_d_*, and K_D_ were not calculated (Figure 4B).

### 3.3. The Site-Specific Substituted rTpiA, Which Does Not Bind to Plasminogen, Does Not Promote Plasminogen Activation

**Figure 5:**
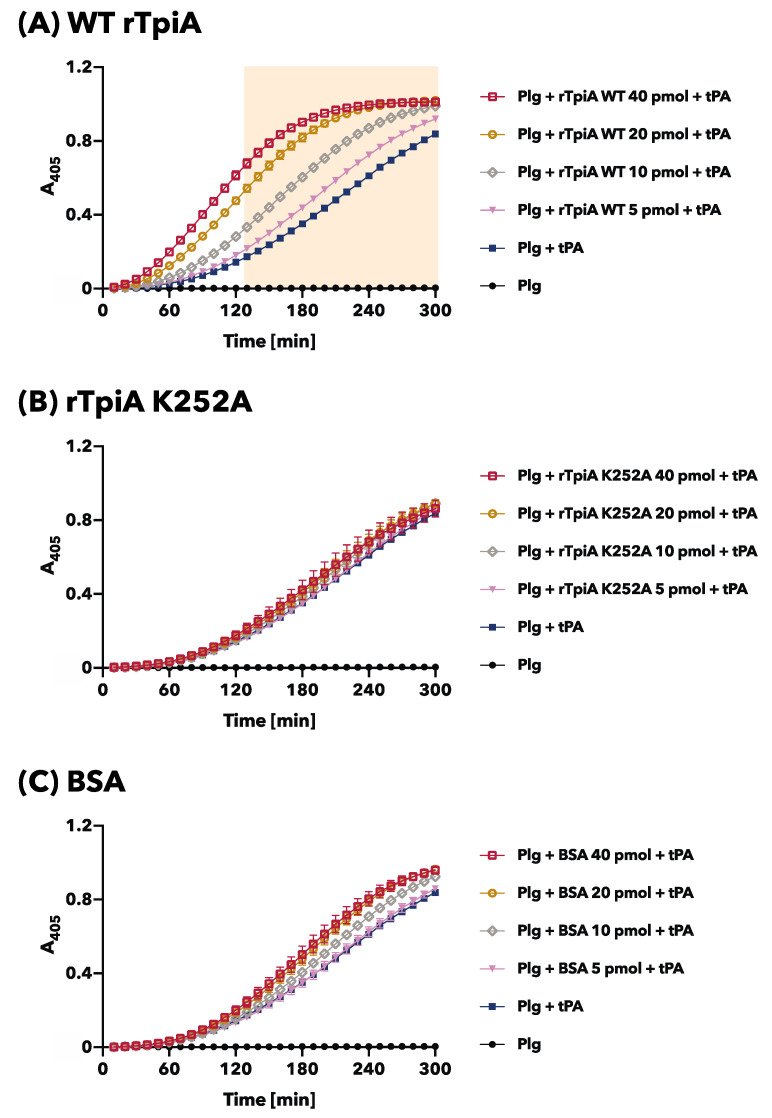
Plasminogen activation assay. Plasminogen was preincubated with wild-type rTpiA (**A**), the site-specific amino acid substituted rTpiA K252A (**B**), or BSA (**C**), and then tPA and the chromogenic substrate were added. The mixtures were incubated, and A_405_ was measured every 10 min. Error bars indicate standard error (*n* = 6). Filled background indicates areas that were significantly higher when preincubated with any dose of wild-type rTpiA compared to Plg + tPA (Dunnett’s multiple comparisons test).

In our previous studies, we discovered that the addition of rTpiA enhanced the activation of plasminogen by the activator tPA [8]. In this study, we investigated the effect of site-specific substitution of rTpiA, which no longer allows binding to plasminogen, on plasminogen activation. Plasminogen was activated by tPA; we observed a time-dependent increase in absorbance as the chromogenic plasmin substrate was degraded (Figure 5). As reported previously, pre-incubation of plasminogen with wild-type rTpiA led to a significant dose-dependent increase in tPA-induced plasminogen activation (Figure 5A). However, when plasminogen was pre-incubated with substituted rTpiA K252A, plasminogen activation by tPA was not promoted (Figure 5B). We used BSA as a control, which did not promote plasminogen activation (Figure 5C). Based on our findings, we conclude that TpiA binds to plasminogen to facilitate plasminogen activation.

## 4. Discussion

After infectious agents infect humans, they cross physical barriers such as human epithelial cells and ECM and enter the bloodstream, becoming invasive and moving to their preferred location in the host tissue while evading the host’s effector system [22,23,24,25]. During this process, infected pathogens often utilize host proteins, including plasminogen [26,27,28]. Human plasminogen is recruited by the pathogen and further activated and converted to plasmin, a proteolytic enzyme that degrades host proteins such as ECM and cell junction proteins to facilitate invasion and colonization [22,29,30].

Human plasminogen has seven structural domains, each with distinct properties. The N-terminal portion of the molecule consists of an activating peptide followed by five repeating homologous triple disulfide-linked peptide regions called Kringles (K1–K5), which are approximately 80 amino acids long [31]. Human plasminogen binds to physiological ligands by recognizing exposed C-terminal lysine residues through the Kringle domain [32,33]. The binding strength of Kringle depends on the nature of the ligand; K1 and K4 show the strongest ligand affinity [31,34,35,36,37], whereas K2 has the weakest affinity [38]. K2 shows a strong affinity for an endopolypeptide derived from the streptococcal plasminogen receptor M protein, which was first found in *S. pyrogenes* [39,40].

Recognition of plasminogen by *S. pneumoniae* is postulated to involve multiple proteins (receptors) that function simultaneously. The expression of these proteins may be regulated by factors such as the phases of growth and infection, tissue localization, and the host immune response. In *S. pneumoniae*, several proteins, including α-enolase [33,41,42,43,44,45,46], glyceraldehyde-3-phosphate dehydrogenase (GAPDH) [47], choline-binding protein E (CbpE) [48], plasminogen- and fibronectin-binding protein B [49], endopeptidase O (PepO) [50], elongation factor Tu (Tuf) [51], phosphate kinase [52], and PspC [53] have been reported to have plasminogen-binding properties. We recently discovered that the proteins triosephosphate isomerase (TpiA) [8], ClpC, and UvrC [18] also have plasminogen-binding properties. In particular, TpiA was shown not only to have high plasminogen-binding affinity but also to promote plasminogen activation. The binding of TpiA to plasminogen is inhibited by EACA, suggesting that lysine residues in TpiA are involved in plasminogen binding [8]. In α-enolase, CbpE, PepO, and Tuf, the lysine residues within the protein molecule are involved in binding to plasminogen [41,43,48,50,51]. However, the plasminogen-binding site is not necessarily located at the C-terminus of the protein molecule. For example, in CbpE, the lysine residue that binds to plasminogen is located approximately one-third the distance from the N-terminus [48]. The plasminogen binding site of phosphate kinases is located at the N-terminus [52]. Of these enzymes, α-enolase is the most studied, in which a plasminogen-binding motif and a C-terminal lysine residue have been identified as being located in the middle. In octameric α-enolase, the binding motif is thought to make a more marked contribution to binding [43].

In this study, we produced 19 mutant recombinants (Figure 1) in which lysine residues in TpiA were replaced with alanine and analyzed their binding to plasminogen. For the preparation of rTpiA, the *Brevibacillus* expression system was used because it is a Gram-positive bacterium, such as *S. pneumoniae*, and because the recombinant protein is easily purified. Three different analyses (far-WB, ELISA, and SPR measurement) showed that rTpiA K252A exhibited significantly lower binding to plasminogen than the other mutant recombinants (Figure 2, Figure 3 and Figure 4). This lysine residue is the C-terminal amino acid of TpiA, and its involvement in binding to plasminogen is consistent with the results of previous studies on pneumococcal α-enolase [41]. Furthermore, we found that TpiA K252A, which lost its ability to bind plasminogen, also lost its ability to promote the activation of plasminogen to plasmin (Figure 5). Therefore, our results suggest that while TpiA promotes activator-induced activation of plasminogen to plasmin, binding to plasminogen is essential for this activation. It will be interesting to see the extent to which TpiA actually contributes to plasminogen binding and its activation when *S. pneumoniae* infects the host. However, because TpiA is an enzyme essential for growth, it was not possible to generate the TpiA gene deletion mutant in *S. pneumoniae*. Instead, it may be possible to generate a strain with TpiA that carries a site-specific amino acid mutation; however, it is not easy to analyze the extent to which TpiA contributes to plasminogen binding because multiple molecules other than TpiA bind to plasminogen.

Note that a previous study has shown that the triosephosphate isomerase of *S. aureus* binds to plasminogen but inhibits plasminogen activation [17]. We compared the amino acid sequence of the triosephosphate isomerase homologue of *S. pneumoniae* with that of other *Streptococcus* species and *S. aureus* and found high identity in *Streptococcus* species (83.2–99.2%), but 58.1% identity in *S. aureus* [8]. This sequence difference may be responsible for the differences in interaction with plasminogen. As with TpiA in *S. pneumoniae*, the C-terminus of triosephosphate isomerase in *S. aureus* is also a lysine residue.

To identify pneumococcal proteins that contribute to infection, we collected bronchoalveolar lavage fluid from a mouse model of pneumococcal pneumonia and identified *S. pneumoniae* proteins using iTRAQ-MS/MS analysis [8]. Only 15 proteins were identified, many of which were endogenous molecules involved in metabolism. Among the proteins identified were α-enolase, GAPDH, Tuf, TpiA, ClpC, and UvrC, all of which have plasminogen-binding properties [8,18]. Although most of these proteins are intracellularly localized and do not have secretory or anchoring signals, they may perform other functions outside the cell or on the cell surface [54,55]. In *S. pneumoniae*, the release of intracellular proteins into the extracellular space is easily achieved because they undergo autolysis during the stationary phase of growth. Despite the fact that the genome of bacteria is small compared to that of eukaryotes and the number of protein molecules produced is small, a single protein molecule can have multiple functions and consequently utilize the functions of host molecules and host energy for infection and invasion, which may be a survival strategy acquired by bacteria through evolution.

## Figures and Tables

**Table 1:** Primers used in this study. The lowercase letters in the forward primers indicate the sequence for replacing the lysine residue with an alanine residue (from AAA to GCT in the DNA sequence). The amino acid substitution positions (e.g., K4A) follow the number of amino acids in the original TpiA sequence of *S. pneumoniae*.

Application		Sequence (5′ to 3′)
K4A	Forward	gctCCATTTATCGCTGGTAACTGG
	Reverse	ACGTGATTTGTCATCGTCATC
K12A	Forward	gctATGAACAAAAATCCAGAAGAAGC
	Reverse	CCAGTTACCAGCGATAAATGG
K15A	Forward	gctAATCCAGAAGAAGCTAAAGCATTC
	Reverse	GTTCATTTTCCAGTTACCAGCG
K21A	Forward	gctGCATTCGTTGAAGCAGTTG
	Reverse	AGCTTCTTCTGGATTTTTGTTC
K30A	Forward	gctCTTCCTTCATCAGATCTTGTTGAAG
	Reverse	TGATGCAACTGCTTCAACG
K56A	Forward	gctGGCTCAAACCTTAAAGTTGCTG
	Reverse	TGCAACAGCAAGAACAGTTGTC
K61A	Forward	CTTgctGTTGCTGCTCAAAACTGCTAC
	Reverse	GTTTGAGCCTTTTGCAACAG
K85A	Forward	gctGAAATCGGTACTGACTACGTTGTTATC
	Reverse	CAAAACTTGTGGGCTAGTTTCAC
K112A	Forward	gctAAAGCAAAAGCAATCTTTGCG
	Reverse	GTTGATATCTTCGTCAGTTTCATGG
K113A	Forward	gctGCAAAAGCAATCTTTGCGAAC
	Reverse	TTTGTTGATATCTTCGTCAGTTTCATG
K115A	Forward	gctGCAATCTTTGCGAACGG
	Reverse	TGCTTTTTTGTTGATATCTTCGTC
K139A	Forward	gctGCTGCTGAATTCGTAGGTGC
	Reverse	ACCAGCTTCGTAAGTTTCAAGTG
K177A	Forward	gctTCAGCTTCACAAGACGATGC
	Reverse	ACCAGTACCGATAGCCCAG
K186A	Forward	gctATGTGTAAAGTTGTTCGTGACGTTG
	Reverse	TTGTGCATCGTCTTGTGAAG
K189A	Forward	gctGTTGTTCGTGACGTTGTAGCTG
	Reverse	ACACATTTTTTGTGCATCGTC
K206A	Forward	gctGTTCGTGTTCAATACGGTGG
	Reverse	GTCTGCGACTTCTTGACCAAAG
K216A	Forward	gctCCTGAAAATGTTGCTTCATACATG
	Reverse	AACAGAACCACCGTATTGAACAC
K252A	Forward	TAgctTAATCAGTAAGTAGC AAGCTTAACAGGATG
	Reverse	CAAAGTCAAGCAAAGCCAAGAAG
K112A, K113A, K115A	Forward	gctgctGCAgctGCAATCTTTGCGAACGGTATG
	Reverse	GTTGATATCTTCGTCAGTTTCATGG
Sequencing	Forward	CGCGATATCAGGATTCGG
	Reverse	CAATGTAATTGTTCCCTACCTGC

## Data Availability

All data are contained within the manuscript.

## Supplementary Materials

The following supporting information can be downloaded at: https://www.mdpi.com/article/10.3390/microorganisms11051198/s1, Figure S1: Original images of SDS-PAGE and CBB staining to detect substituted rTpiA proteins. Figure S2: Binding activity of wild-type rTpiA and the site-specific amino acid substitution rTpiA K252A to plasminogen measured by SPR (second and third experiments).
