# Supplementary material for: C-Terminal Lysine Residue of Pneumococcal Triosephosphate Isomerase Contributes to Its Binding to Host Plasminogen
Source: Microorganisms. 2023 May 4;11(5):1198. doi: 10.3390/microorganisms11051198 (PMC10221034; doi:10.3390/microorganisms11051198)
Supplement: Supplementary file 1 [file microorganisms-11-01198-s001.zip › microorganisms-2318848-supplementary.pdf]

Supplementary Figures

C-terminal lysine residue of pneumococcal triosephosphate isomerase contributes to its binding to host plasminogen

Satoru Hirayama [1], Takumi Hiyoshi [1,2,3], Yoshihito Yasui [1,2], Hisanori Domon [1,3] and Yutaka Terao [1,3,*]

[1] Division of Microbiology and Infectious Diseases, Niigata University Graduate School of Medical and Dental Sciences, Niigata, Japan
[2] Division of Periodontology, Niigata University Graduate School of Medical and Dental Sciences, Niigata, Japan
[3] Center for Advanced Oral Science, Niigata University Graduate School of Medical and Dental Sciences, Niigata, Japan
***** Correspondence: terao@dent.niigata-u.ac.jp

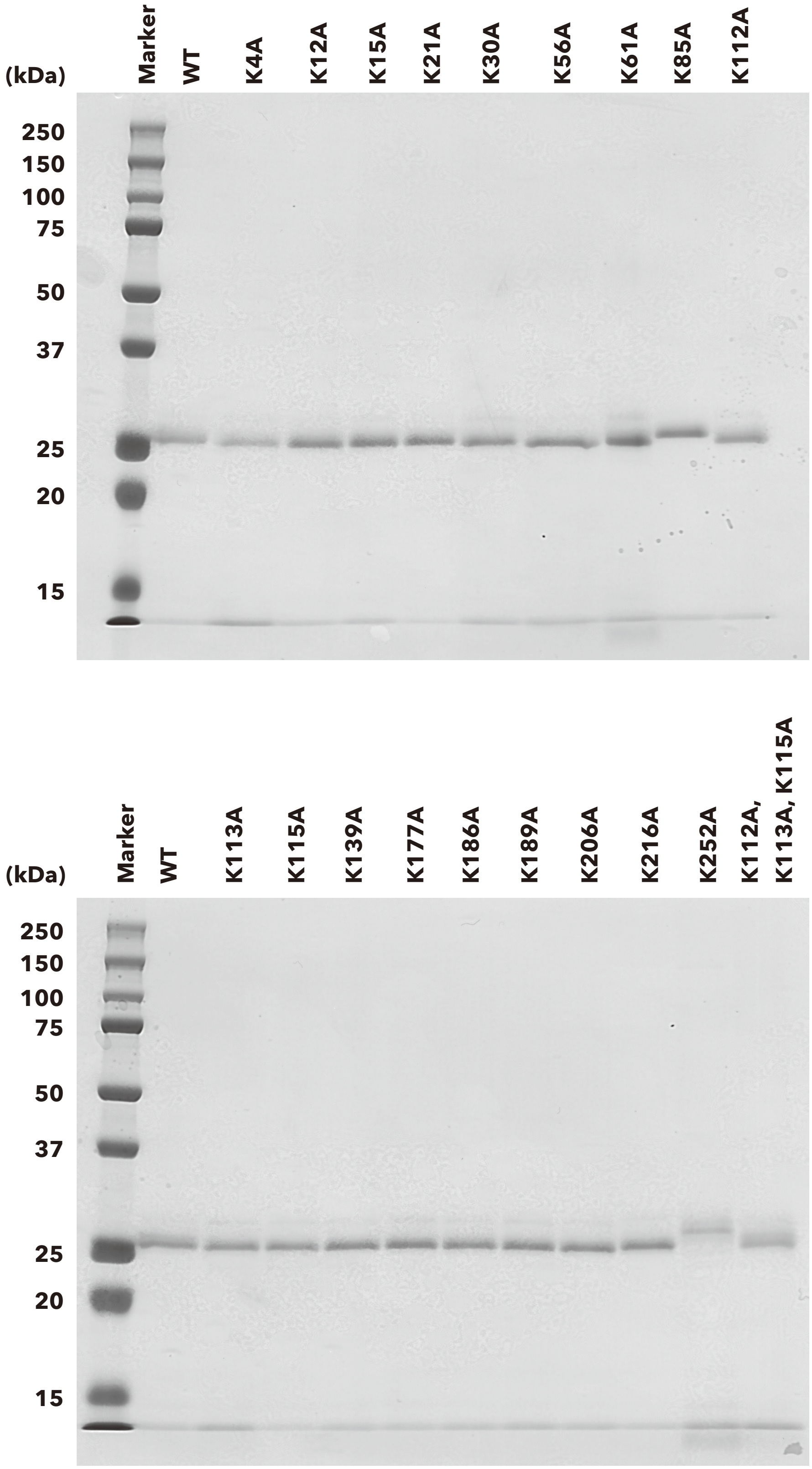

Supplementary Figure S1. Original images of SDS-PAGE and CBB staining to detect substituted rTpiA proteins. Unprocessed images of the images shown in Figure 2A.

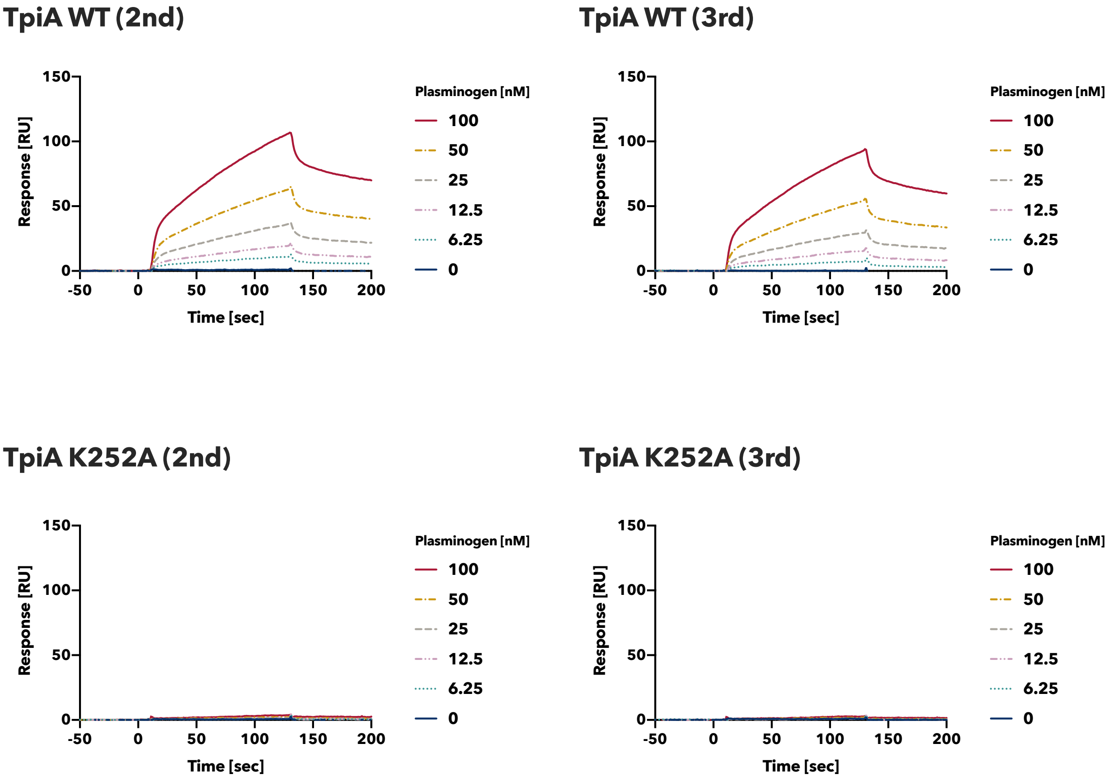

Supplementary Figure S2. Binding activity of wild-type rTpiA and the site-specific amino acid substitution rTpiA K252A to plasminogen measured by SPR (second and third experiments). The first experiment is shown in Figure 4.
